# Approach and Avoidance of Emotional Faces in Happy and Sad Mood

**DOI:** 10.1007/s10608-012-9436-9

**Published:** 2012-02-08

**Authors:** Janna N. Vrijsen, Iris van Oostrom, Anne Speckens, Eni S. Becker, Mike Rinck

**Affiliations:** 1 Department of Psychiatry, Radboud University Nijmegen Medical Centre, PO Box 9101, 6500 HB Nijmegen, The Netherlands; 2 Behavioural Science Institute, Radboud University Nijmegen, Nijmegen, The Netherlands

**Keywords:** Mood, Approach, Avoidance, Depression, Facial expressions

## Abstract

Since the introduction of the associative network theory, mood-congruent biases in emotional information processing have been established in individuals in a sad and happy mood. Research has concentrated on memory and attentional biases. According to the network theory, mood-congruent *behavioral tendencies* would also be predicted. Alternatively, a general avoidance pattern would also be in line with the theory. Since cognitive biases have been assumed to operate strongly in case of social stimuli, mood-induced biases in approach and avoidance behavior towards emotional facial expressions were studied. 306 females were subjected to a highly emotional fragment of a sad or a happy movie, to induce either a sad mood or a happy mood. An Approach-Avoidance Task was implemented, in which single pictures of faces (with angry, sad, happy, or neutral expression) and non-social control pictures were presented. In contrast to our expectations, mood states did not produce differential behavioral biases. Mood-congruent and mood-incongruent behavioral tendencies were, however, present in a subgroup of participants with highest depressive symptomatology scores. This suggests that behavioral approach-avoidance biases are not sensitive to mood state, but more related to depressive characteristics.

## Introduction

According to network models of emotion (e.g., Bower 1981), individuals in a positive or negative mood show preferred cognitive processing of mood-congruent stimuli, for instance, by automatically attending to mood-congruent stimuli and remembering it better. Indeed, there is strong evidence for biased memory processes, favoring memory for negative information in sad mood and for positive information in neutral or happy mood (Matt et al. 1992). Moreover, depression (in remission) and dysphoria are also characterized by cognitive biases (Mathews and MacLeod 2005; Williams et al. 1997). In sub-clinical samples, biases generally become more pronounced when individuals are primed with a negative mood induction (Segal and Ingram 1994). Taken together, this suggests that mood-congruent processing occurs in both clinical depression and sub-clinical negative affective states, and in naturally occuring moods as well as in experimentally induced states. Preferred processing of negative information by sad individuals may be a risk factor for maladaptive levels of negative mood and even clinical depression (Compton 2000). Gaining more insight into mood-congruent processing in sad mood thus seems of importance.

The network theory, together with the extensive research conducted on mood-congruent biases, suggests that such tendencies might prevail in other automatic processes as well. Therefore we aimed to study a very basic motivated behavioral tendency, namely automatic approach-avoidance behavior. Individuals generally aim to approach a reference stimulus when a positive outcome or affect is expected and avoid information with a possible negative outcome such as negative affect (Carver et al. 2008). Behavioral approach-avoidance has not been studied so far with regard to mood, however (Trew 2011). Mood is a strong motivator for our automatic behaviors. In fact, sad mood is characterized by behavioral avoidance, which seems to be especially salient in the social domain (Ottenbreit and Dobson 2004). Studying social avoidance behavior in sad mood is important because this behavior generally reduces satisfying experiences, and therefore maintains a sad mood state and might even contribute to the development of clinical depression (Ferster 1973).

For humans, social information communicated through facial expressions bears high social relevance (Lee et al. 2008). Moreover, biases have been assumed to operate most strongly in case of social or interpersonal stimuli, like faces (Hammen 1992). Therefore, we studied the effects of mood on approach-avoidance tendencies for facial stimuli.

Automatic behavioral approach and avoidance tendencies to different facial expressions were studied using the Approach Avoidance Task (AAT; Rinck and Becker 2007) in social anxiety (Heuer et al. 2007; Lange et al. 2008), where socially anxious individuals tended to avoid angry as well as happy faces more than control participants did. The AAT is based on the finding that stimulus valence is related to behavioral approach and avoidance inclinations. Positively valenced stimuli produce immediate approach tendencies, whereas negative stimuli produce avoidance tendencies (Chen and Bargh 1999). In the present AAT, we used facial stimuli expressing different emotions (namely sad, happy, angry, and neutral). Sad as well as angry faces were included to provide an indication of whether avoidance behavior is mood-specific (i.e., only towards sad faces) or more general.

We induced a sad mood state in half and a happy mood in the other half of the participants. The study aimed at providing novel insights into emotion-specific behavioral tendencies of individuals in a sad mood. Based on before mentioned theories, several behavioral tendencies would be predicted for both mood states. Individuals in a sad mood could be expected to approach the negative and avoid mood-incongruent facial expressions and individuals in a positive mood could be expected to approach happy and neutral faces while avoiding negative ones. Thinking in lines of the network models and (biological) theories of motivational orientation (e.g. Strack and Deutsch 2004; Davidson 1992, 1993), one could also argue that sad mood would facilitate avoidance tendency, while positive mood would be characterized by a general approach tendency, regardless of the valence of the reference object. Sad mood functioned as an analogue for more clinical levels of negative mood, as in depression, and also as a prime for biased processing. Stronger behavioral tendencies were therefore predicted in more vulnerable participants with a higher level of depressive symptomatology after the mood induction.

## Methods

### Participants

We tested 318 female undergraduate students of Radboud University Nijmegen. They were randomly subjected to either a sad or a happy mood induction. Individuals who were taking antidepressant medication or sleeping aids were excluded, resulting in a sample of 158 individuals in a happy mood (HMs) and 148 participants in a sad mood (SMs). At baseline, SMs and HMs did not differ with regard to depressive symptoms; mean scores were 5.1 (SD = 5.0) and 5.9 (SD = 5.4), respectively, on the Beck Depression Inventory (BDI-II; Beck et al. 1996), and 5.9 (SD = 6.1) and 6.7 (SD = 8.1) on the depression subscale of the Symptom Checklist (SCL-90; Derogatis et al. 1973). Neither did SMs and HMs differ with regard to overall level of psychopathology on the SCL-90; mean scores were 27.5 (SD = 24.5) for SMs and 31.0 (SD = 31.4) for HMs. The two experimental groups were comparable with regard to age (mean age SMs = 21.0, SD = 3.5, and mean age HMs = 21.0, SD = 3.0).

More vulnerable populations, with a higher level of depressive symptomatology, were expected to have stronger behavioral tendencies. Therefore, based on their total BDI scores, the sample was divided into three groups, each consisting of as close to 1/3 of the participants as possible. The lowest-BDI group had a mean BDI score of 1 (SD = 0.9, range 0–2) and consisted of 29% of the participants, N = 92. The medium-BDI group had a mean BDI score of 4.3 (SD = 1.1, range 3–6) and contained 41% of the participants, N = 118. The highest-BDI group contained 30% of the participants, with a mean BDI score of 11.3 (SD = 5.5, range 7–30), N = 96. Of these, 25 participants had a total BDI score above 12, indicating clinical dysphoria or depression (Dozois et al. 1998).

### Materials and Apparatus

Color photos of the faces of 10 individuals (5 female, 5 male) from the Radboud Faces Database [RaFD; Langner et al. (2010)] were used. For each individual, an angry, sad, happy, and neutral expression was selected. Additionally, 10 control pictures displaying the same chessboard pattern were included. By placing either a purple or a blue color filter over the picture, two versions of each of the 50 pictures were created, resulting in 100 picture stimuli. An additional set of 18 mixed social and control pictures served as practice trials. A computer screen with a resolution of 1,024 × 768 pixels and a “Logitech Attack 3” joystick were employed.

A ‘zooming-effect’ was used to create the visual impression that the pictures were actually being approached or avoided: The pictures grew and seemed to come closer upon pulling of the joystick and to shrink and move away upon pushing it. When the maximum possible movement of the joystick was made, the picture would disappear. The time from initiation of the trial to disappearance of the picture was automatically recorded and used as the participant’s reaction time (RT; see Rinck and Becker 2007, for details).

### Procedure

The participant first completed the BDI, the SCL-90, and some questions about biographical data. Then the mood induction started. It consisted of a 12-min fragment from an emotional movie (‘Happy Feet’ for happy mood and ‘Sophie’s Choice’ for sad mood). The participant was instructed to watch the movie, listen to the sounds of the movie, and to let the emotionality of the movie influence her mood. The participant was asked to rate her current mood state using a computerized visual analogue scale that ranged from -10 (indicating saddest mood) to 10 (indicating happiest mood) at baseline and after the mood induction. She continued with the AAT, for which instructions appeared on the computer screen. She was instructed that pictures of faces would be appearing on the screen, one by one, and to either push the picture away or pull it towards herself in order to make the picture disappear as soon as possible. Pictures with a blue filter had to be pushed away, while purple pictures had to be pulled.

## Results

The mood induction procedure was successful. A *t* test conducted on these data points indicates that SMs (M = 5.4, SD = 3.1) and HMs (M = 4.5, SD = 3.4) did not differ on mood state at baseline, *t*(80) = 1.19, *P* = .23. Due to a programming error, the baseline mood state ratings of only 82 participants were available. SMs (M = −0.4, SD = 4.2) and HMs (M = 6.3, SD = 2.4) differed significantly on mood after the mood induction, *t*(300) = 16.91, *P* < .001. Within the lowest-BDI group, the mood groups differed marginally significant at the baseline mood rating (SMs M = 7.1, SD = 1.2, HMs M = 6.1, SD = 1.7), *t*(24) = 1.81, *P* = .08. This difference was non-significant for the medium-BDI group (SMs M = 5.6, SD = 2.29, HMs M = 4.8, SD = 2.5), *t*(26) = .77, *P* = .45, as well as for the highest-BDI group (SMs M = 3.4, SD = 3.5, HMs M = 3.0, SD = 4.6), *t*(24) = .28, *P* = .78. SMs and HMs differed on mood after the mood induction in all subgroups: SMs M = 0.4, SD = 4.0 and HMs M = 6.6, SD = 2.0 with *t*(90) = 9.08, *P* < .001 the lowest-BDI group, SMs M = −1.0, SD = 4.4 and HMs M = 6.6, SD = 1.9 with *t*(115) = 12.46, *P* < .001 the medium-BDI group, and SMs M = −0.5, SD = 4.4 and HMs M = 5.8, SD = 3.2 with *t*(91) = 8.01, *P* < .001 the highest-BDI group. When comparing baseline to post induction mood ratings in the highest-BDI group, a significant decrease of mood in SMs, *F*(1,13) = 17.62, *P* < .05, *f* = 1.16, and an increase of mood in HM, *F*(1,11) = 19.81, *P* < .05, *f* = 1.34, was uncovered.

The top and bottom 1% of trial RTs as well as participants with a mean overall RT of >1,000 ms and/or >20% errors (6 participants in total) were deleted after outlier analyses. Error rates were low, on average less than 5%. Mean RT scores were then computed for each participant for each combination of picture type (angry, sad, happy, neutral, or control) and response direction (push, pull) for correct responses only. Next, AAT effect scores were computed for each participant by subtracting the mean RT of the pull trials from the RT of the corresponding push trials, separately per picture type. Negative scores mean that the participant was relatively faster pushing the pictures away than pulling them closer.

SMs (mean = 615, SD = 90) and HMs (mean = 612, SD = 90) did not differ in mean overall reaction time, neither did the two groups differ in approach-avoidance tendencies towards the non-social control pictures (mean = −20 (SD = 84) for SMs and mean = −14 (SD = 97) for HMs). No Mood × Depressive Symptoms interaction was found for the non-social control stimuli, *F*(2,300) = 0.64, *P* = .53, *f* = 0.06.

The AAT effect scores for correct responses depending on group and facial expression are shown in Table 1. The 2 (Mood State) × 3 (Depressive Symptoms) × 4 (Facial Expression) mixed analyses of variance (ANOVA) for repeated measure of the AAT effect scores, with the first two factors varying between participants, yielded a main effect of Facial Expression, *F*(3,298) = 7.20, *P* < .001, *f* = 0.15. Here angry, happy, and neutral faces were all avoided more than sad faces. This effect was observed in the lowest-, medium-, and highest-BDI groups, for whom significant or marginally significant main effects of Facial Expression were found, *F*(3,88) = 4.48, *P* < .05, *f* = 0.15 for the group lowest, *F*(3,114) = 2.17, *P* = .09, *f* = 0.15 for medium, and *F*(3,92) = 4.01, *P* < .05, *f* = 0.15 for the group highest on BDI.

**Table 1:** AAT effect scores (push minus pull) with SDs depending on mood, level of depressive symptoms, and facial expression

Facial expression	BDI group	Group	
		SM	HM
Angry	Lowest	−12 (66)	−35 (84)
	Medium	−30 (79)	−9 (67)
	Highest	12 (77)	−20 (79)
	Total	−11 (75)	−19 (76)
Sad	Lowest	19 (84)	12 (72)
	Medium	5 (93)	0 (86)
	Highest	−4 (70)	19 (89)
	Total	7 (84)	10 (83)
Happy	Lowest	4 (104)	−20 (91)
	Medium	−14 (85)	4 (94)
	Highest	−37 (83)	−16 (76)
	Total	−14 (92)	−9 (88)
Neutral	Lowest	−24 (82)	4 (99)
	Medium	−29 (93)	−14 (70)
	Highest	−21 (86)	−23 (71)
	Total	−25 (88)	−12 (79)

**Fig. 1:**
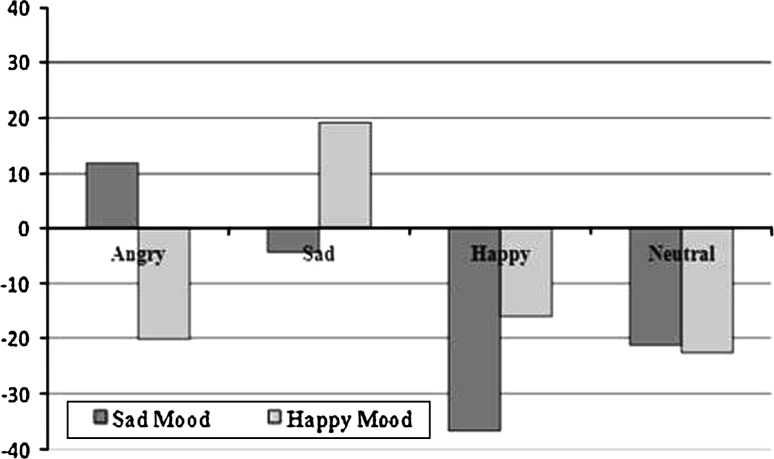
AAT effect scores depending on mood and facial expression in the group highest on depressive symptoms. Positive score represent relative approach and negative scores relative avoidance

The Mood × Facial Expression interaction yielded non-significant results, *F*(3,298) = 1.80, *P* = .14, *f* = .14, indicating that mood state did not seem to affect automatic behavioral tendencies towards the different facial expressions. The Mood × Depressive Symptoms interaction was also non-significant, *F*(2,300) = 0.96, *P* = .39, *f* = 0.08. However, the 2 × 3 × 4 ANOVA yielded a significant three-way interaction, *F*(6,596) = 2.20, *P* < .05, *f* = 0.15. Additional analyses revealed that this interaction was mostly driven by the mood effects in the highest-BDI group: For these participants, the Mood × Facial Expression interaction was marginally significant, *F*(3,92) = 2.42, *P* = .07, *f* = 0.15 (see Fig. 1). Here, angry faces were avoided more by HMs than by SMs, while the reverse was true for sad faces. Happy and neutral facial expressions seem to be avoided by both mood groups within the highest-BDI participants.

HMs were more avoidant of angry expressions than SMs, *t*(94) = 2.00, *P* < .05, while no differences were found for the other expressions, *t*(94) = 1.42, *P* = .16 for sad, *t*(94) = 1.28, *P* = .20 for happy, and *t*(94) = 0.08, *P* = .94 for neutral faces. Within HMs highest on BDI, angry faces were avoided more than sad faces, *t*(52) = 2.25, *P* < .05. Angry faces were also avoided more than happy, *t*(52) = 2.40, *P* < .05, and neutral faces, *t*(52) = 2.56, *P* < .05*.* The other expressions elicited similar responses. For SMs in the highest-BDI group, happy faces evoked more avoidance tendencies than angry faces, *t*(42) = 2.65, *P* < .05, as well as sad faces, *t*(42) = 2.04, *P* < .05. Neutral expressions were also avoided more than angry expressions, *t*(42) = 2.19, *P* < .05. The remaining expressions elicited similar reactions in SMs.

## Discussion

As expected, the results of the current study suggest that some facial expressions are avoided while others are more easily approached. However, no indication of mood-congruent biases or more global approach or avoidance tendencies were found. Since mood-congruent biases as well as avoidance behavior have often been observed most strongly in clinical samples, we expected more pronounced effects of the mood induction at higher levels of depressive symptoms. Here, the mood induction was expected to function as a prime for biased processing. Indeed, an indication of mood-congruent as well as incongruent behavioral biases was found in the group scoring highest on depressive symptomatology. The mood induction seems to elicit processing biases (in line with e.g. Joormann and Siemer 2004). In contrast, the behavior tendencies elicited by non-social control stimuli were similar in both moods, even in the sub-group highest on depressive symptomatology. This demonstrates that only emotional social stimuli elicit differential behavioral responses, and presumably the strongest in more vulnerable individuals.

In the group with the highest depressive symptomatology, SMs tended to avoid happy faces the most. Neutral expressions were also avoided more than angry faces. These results corroborate nicely with associative-network theories. In line with earlier findings on mood-congruence, happy and neutral faces are incompatible with SMs’ mood state, and those expressions are therefore avoided most. The finding that sad and angry faces are avoided less or even approached, signifies that both negative expressions are considered most mood-congruent for this group. So not only mood-specific stimuli, but negatively valenced stimuli in general, seem to be congruent with a sad mood.

For HMs in the group with the highest depressive symptomatology, however, the picture is not as clear. This group tended to avoid angry, happy, and neutral faces more than sad expressions. The avoidance of angry faces might be evolutionary-based as an angry expression is generally considered aversive and threatening (Marsh et al. 2005). Strikingly, HMs scoring highest on the BDI generally approached sad faces. This was unexpected, because according to mood-congruence, happy individuals should approach happy faces and avoid sad ones. If sad faces do indeed elicit approach, then perceivers might perhaps experience feelings of rapport as it encourages the formation of social bonds. Indeed, some evidence suggests that sadness elicits not only the desire for affiliation, but also emphatic behavior (e.g., Batson et al. 1981). Individuals who received a happy mood induction probably have the capacity and drive to act emphatically and approach sad faces. As an addition to this paradigm, we would therefore be interested in testing whether HMs explicitly report more empathic experiences than SMs.

Neutral faces were avoided equally by SMs and HMs in the group with the highest depressive symptomatology. Apparently, neutral facial expressions were regarded as mood-incongruent by both mood groups. Since the mood induction was successful, it seems plausible that for individuals in an induced sad mood, only negative faces were mood-congruent. The same would hold for individuals in a pronounced happy mood: Only clearly positive expressions would be conceived as congruent. Taken together, behavioral biases seem to be characterized by mood-congruent and incongruent processing. Since no evidence was found for a general avoidance pattern in sad mood or a general approach tendency happy mood, it appears that the valence of the stimulus affects the automatic behavioral tendencies. Furthermore, the results might indicate that behavioral biases are more related to depressive symptomatology than to state mood effects and it also indicates that such tendencies might not be as sensitive to mood differences as cognitive biases are. Such conclusions can however not be drawn based on these finding, underscoring the need for further investigation.

Although the current findings must be interpreted with care, since only a specific segment of behavioral avoidance was assessed in a controlled setting, behavioral avoidance of positive interpersonal information might be a dysfunctional automatic tendency in dysphoria and depression. Avoiding positive social experiences may keep vulnerable sad individuals from feeling better and hence help maintain their current mood state and perhaps even worsen it (Compton 2000). Avoidance behavior holds a central role in mood disorders and contributes to the development and maintenance of depression (Ferster 1973). One may hypothesize that specifically the avoidance of positive social information (e.g., happy faces) by vulnerable sad individuals is a maladaptive automatic tendency which could be one of the determinants of the eventual development of depression.

A few words of caution seem appropriate, however. First, gender effects were not taken into account, since an all female sample was tested. This might limit the study’s generalizability, because men might exhibit different behavioral approach-avoidance tendencies than women. For future studies, it would be recommended to include male subjects so potential gender influences can be studied. Secondly, although induced sad mood state is often thought of and used as an analogue for depression, it is not perfectly comparable to depressed mood. Besides sad mood, many other deficits characterize depression which are not as salient in individuals in which sad mood is experimentally induced. Furthermore, only a portion of the group highest on depressive symptomatology had BDI scores indicating clinical levels of dysphoria or depression. In order to make any statements about behavioral tendencies in depression, this study first needs to be replicated in a depressed patient sample, preferably including a group without mood manipulation. As for now, we can only speculate that such studies will yield more compelling results. On the other hand, the fact that the task revealed results even in a sub-clinical sample suggests that automatic behavioral tendencies might be an important factor in the maintenance of depression.

In sum, the current study provides an exploration of the automatic avoidance tendencies in response to social information in sad and happy mood. Induced mood states did not seem to result in mood-congruent behavioral tendencies. When in addition to mood states depressive symptomatology was taken into account, however, specific automatic approach and avoidance tendencies did become apparent in the most vulnerable group. No general pattern of approach of avoidance was discovered. This signifies that, compared to memory and attention biases, behavioral biases may be more related to depressive characteristics in combination with (or exacerbated by) a mood manipulation than to transient moods per se. Hence, this study stresses the need for studying approach-avoidance behavior in clinical depression.
